# Building a locally diploid genome and transcriptome of the diatom *Fragilariopsis cylindrus*

**DOI:** 10.1038/sdata.2017.149

**Published:** 2017-10-10

**Authors:** Pirita Paajanen, Jan Strauss, Cock van Oosterhout, Mark McMullan, Matthew D. Clark, Thomas Mock

**Affiliations:** 1 Department of Cell and Developmental Biology, John Innes Centre, Norwich Research Park, Norwich NR4 7UH, UK; 2 European Molecular Biology Laboratory (EMBL) Hamburg, c/o German Electron Synchrotron (DESY), Notkestraße 85, 22607 Hamburg, Germany; 3 School of Environmental Sciences, University of East Anglia, Norwich Research Park, Norwich NR4 7TJ, UK; 4 Earlham Institute, Norwich Research Park, Norwich NR4 7HU, UK

**Keywords:** DNA sequencing, Genome assembly algorithms, Water microbiology, Genome

## Abstract

The genome of the cold-adapted diatom *Fragilariopsis cylindrus* is characterized by highly diverged haplotypes that intersperse its homozygous genome. Here, we describe how a combination of PacBio DNA and Illumina RNA sequencing can be used to resolve this complex genomic landscape locally into the highly diverged haplotypes, and how to map various environmentally controlled transcripts onto individual haplotypes. We assembled PacBio sequence data with the FALCON assembler and created a haplotype resolved annotation of the assembly using annotations of a Sanger sequenced *F. cylindrus* genome. RNA-seq datasets from six different growth conditions were used to resolve allele-specifc gene expression in *F. cylindrus.* This approach enables to study differential expression of alleles in a complex genomic landscape and provides a useful tool to study how diverged haplotypes in diploid organisms are used for adaptation and evolution to highly variable environments.

## Background & Summary

How extreme and highly variable environmental conditions impact the evolution and adaptation of eukaryotic organisms has not been extensively studied, yet. Our recent work on the diploid polar diatom *Fragilariopsis cylindrus*^1^, which has successfully adapted to the sea-ice environment of the Southern Ocean, showed that approximately 25% of its diploid genome is characterized by highly diverged haplotypes that are interspersed throughout its otherwise homozygous genome. Alleles from these diverged haplotypes were differentially expressed across variable environmental conditions. Rather than facilitating an adaptive response through the differential expression of sub- or neo-functionalised genes^2^, the allelic variation within genes of this polar diatom is differentially expressed^1^, which we refer to as environment-dependent Differential Allelic Expression (DAE). Genes with the most pronounced DAE had the largest ratio of non-synonomous to synonomous nucleotide substitutions (d_N_/d_S_), suggesting a correlation between diversifying selection and allelic differentiation, hence a potential role of the divergent alleles for adaptation to environmental fluctuations (e.g., sea-ice formation and melting) in the Southern Ocean.

The most challenging part of this work was to build the locally diploid genome and transcriptome as the genome was randomly interspersed with diverged haplotype contigs assembled with ARACHNE^3^ based on Sanger sequencing. To resolve the exact location of these diverged haplotype contigs, we used the diploid aware FALCON assembler^4^ for long PacBio sequence reads (Data Citation 1). To resolve genes that have different haplotypes, we lifted the annotation off the ARACHNE assembly, and mapped it to the FALCON assembly, which enabled us to differentiate between alleles and paralogs.

To study gene expression, RNA-seq datasets from six different experimental treatments were created, using samples from (1) optimal growth, (2) freezing temperatures, (3) elevated temperatures, (4) elevated carbon dioxide, (5) iron starvation, and (6) prolonged darkness (Table 1) (Data Citation 2).

**Figure 1:**
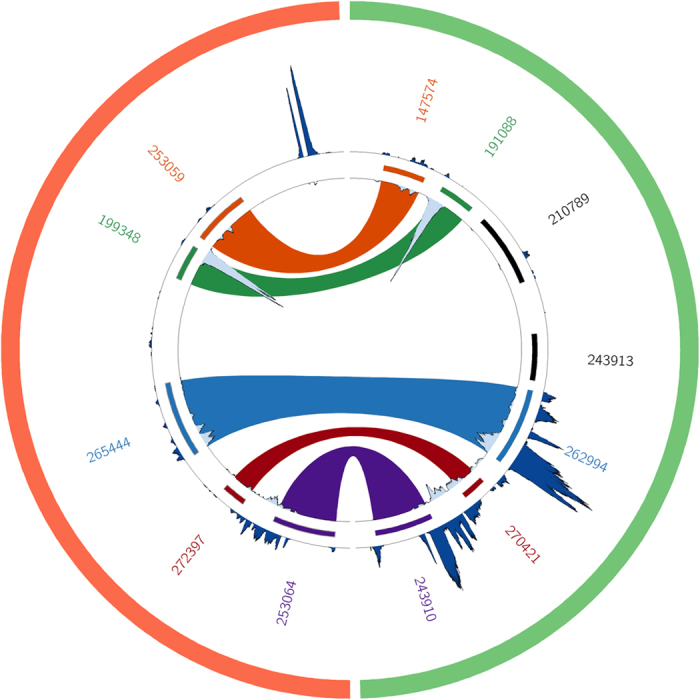
Circular visualisation^7^ of the haplotype resolved expression of allelic variant pairs based on the FALCON assembly. The green semicircle is the primary scaffold 000008F|quiver:437,634–457,643, and the red semicircle is the alternate scaffold on 000008F-002-01|quiver:36,493–56,493. The allelic pairs are joined and colour coded, and annotated using the Joint Genome Institute (JGI) protein identifiers. The dark blue outer histogram indicates the expression level on the prolonged darkness experiment and the light blue inner histogram indicates the expression level under optimal growth conditions. The allelic pair with JGI protein identifiers 262994 and 265444 (blue ribbon; annotated as long-chain acyl-CoA synthetase involved in fatty acid β-oxidation^1^) is differentially expressed.

These datasets were mapped onto the FALCON assembly to resolve haplotype-specifc gene expression in the complex landscape of the *F. cylindrus* genome (Fig. 1).

## Methods

### Diatom cell culture for DNA extraction for genome assembly with FALCON

*Fragilariopsis cylindrus* (Grunow) Krieger CCMP1102 was originally isolated from Southern Ocean seawater samples taken at 64.08° S 48.7033° W (South Orkney Island Research Cruise, Station 12, 16th March 1979) (ref. 4) and obtained from the National Centre for Marine Algae and Microbiota (NCMA, East Boothbay, ME, USA). Cell cultures were maintained and grown at +4 °C in f/2 medium using standard microalgae culturing techniques^5^ to provide material for DNA extraction. Axenic cultures were single cell sorted into 96 well plates (InFlux flow cytometer, BD Biosciences, San Jose, CA, USA) and one of these uniclonal and axenic cultures was used for whole-genome sequencing. Genomic DNA for genome sequencing was extracted using a cetyltrimethylammonium bromide (CTAB) method modified from Doyle and Doyle^6^.

### DNA library preparation and PacBio sequencing

For genome assembly, we created a 20 kb fragment length library and sequenced 3 SMRT cells with the P6C4 chemistry on a PacBio RSII instrument (Earlham Institute, Norwich, UK). All sequencing reads were collected using standard PacBio single-molecule real-time (SMRT) sequencing protocols (Data Citation 1). The total yield was 1.37 Gb of data. The final N50 of read length varied between 8,215 to 8,898 bp. We also created a 4 kb insert size library and sequenced 4 SMRT cells with the P6C4 chemistry on a PacBio RSII instrument, to generate more accurate Read of Insert (RoI) consensus sequences from multi-pass reads of the same library molecules (Data Citation 1). The total amount of data was 3.85 Gb and the N50 ranged from 2,558 to 2,680 bp between the SMRTcells. We combined the data from these 7 SMRT cells and the total coverage of the raw data was 87x. After filtering the shortest reads, we had 3.8 Gb of data which gave 63x coverage.

### Genome assembly with FALCON

We used the diploid aware PacBio assembler, *FALCON* 0.3.0 (ref. 4) to assemble the *F. cylindrus* genome.

Raw subreads fasta files the 7 SMRTcells were used as input data for *FALCON* which was run with default parameters, except the minimum cut off for long reads we set to 2,000 bp. See, fc_di_UV.cfg, (github.com/paajanen/FC_scientific_data/) specifying all the parameters used for the Falcon assembly. We note that the length of the genome was not a parameter that the assembler required.

The assembly was submitted in an environment containing *PYTHON* 2.7.10 using the command fc_run.py fc_di_UV.cfg.

**Figure 2:**
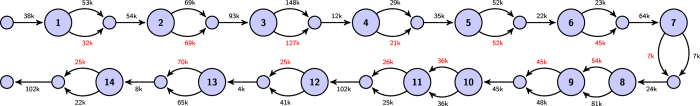
Simple string graph illustration of the highly diverged regions interspersed with homozygous regions for scaffold 000002F. Highly diverged parts of the genome are split by the FALCON assembler into two diverged haplotypes creating bubbles. There are 14 locations with alternate haplotypes. The primary scaffold is obtained, when the graph is traversed following the edges with scaffold lengths (in bp) marked in black, while the alternate haplotypes have their lengths marked in red. These lengths may vary due to natural structural variation, such as tandem duplications, which we have observed elsewhere in the assembly, making the lengths different as is possible for any diploid organism.

The output of the *FALCON* assembler was divided into two parts. The haploid assembly resulted in primary contigs from which we deduced a genome size of 59.7 Mb. However, the assembler also produced alternate contigs, which represent two diverged haplotypes for those regions (Fig. 2). The assembly metrics of the two sets of contigs are shown in Table 2.

We used *QUIVER* to polish the PacBio assembly using part of the *SMRTANALYSIS* 2.3.0p5 (pacb.com) pipeline using default settings and the combined PacBio data from 7 SMRT cells in native h5 format of PacBio. In preparation for polishing, we had to rename the scaffolds from the *FALCON* assembly, by concatenating the fasta names, using a custom script, called rename_falcon_github.py (github.com/paajanen/FC_scientific_data/). We uploaded both the renamed primary contigs and the renamed alternate contigs separately as a reference the polishing using referenceUploader  --skipIndexUpdate  -c -n "reference" -p /path/to/working_directory -f /path/to/reference.fasta  --saw="sawriter -blt 8 -welter" --samIdx="samtools faidx"  --jobId="Anonymous"    --verbose. The polishing step was submitted with command smrtpipe.py --params=polishing_params.xml and the parameter file polishing_params.xml is available at github.com/paajanen/FC_scientific_data/.

### Annotation of the PacBio genome

We extracted the gene models from the annotation provided by the Joint Genome Institute (JGI) (Fracy1_GeneModels_FilteredModels1_nt.fasta from the JGI website at http://genome.jgi.doe.gov/pages/dynamicOrganismDownload.jsf?organism=Fracy1) of *F. cylindrus* and mapped them to the FALCON assembly using GMAP v 20160816 (ref. 8) with the commands gmap_build -D./ -d database assembly.fasta gmap -D /annotation/ -d database -f samse -n 0 -t 16 Fracy1_GeneModels_FilteredModels1_nt.fasta > mapped.sam 2 > mapped.log

We used samtools 1.3 (ref. 9) to create a bam alignment file, and used the commands samtools view -bS, samtools sort and samtools index to sort and index it. From the bam file we created a gtf annotation file by using the tool bam2gtf.py from Mikado version 1.0.0 (https://github.com/lucventurini/mikado).

### Fosmid sequencing and assembly

The fosmids were Sanger sequenced from paired end sub clones and then computational and experimentally finished using Consed^10^ with direct primers walking on sub clones (Data Citation 3).

### Cell culture for RNA-seq experiments

*Fragilariopsis cylindrus* was grown and maintained in filter-sterilised (0.2 μm pore size) Aquil artificial seawater medium^11,12^, which had been adjusted to pH 8.1–8.4 prior to use. Cultures were grown at 4 °C under continuous illumination at a photon flux density of 35 μmol photons m^−2^ s^−1^ (QSL 2101, Biospherical Instruments Inc., San Diego, CA, USA) from cool white fluorescent tubes using a temperature and light controllable incubator (RUMED light thermostate type 1301, Rubarth Apparate GmbH, Laatzen, Germany). Cell cultures were handled under strict sterile conditions and potential bacterial contamination was eliminated as stock cultures were subjected to a multi-antibiotic treatment with ampicillin (50 μg ml^−1^), gentamycin (1 μg ml^−1^), streptomycin (25 μg ml^−1^), chloramphenicol (1 μg ml^−1^) and ciprofloxacin (10 μg ml^−1^) (ref. 13) for up to 7 days.

Fluorescence microscopy combined with 4',6-diamidino-2-phenylindole (DAPI) fluorescent nucleic acid staining was used to confirm axenic cultures before the beginning of culture experiments.

Experimental batch cultures were grown in three biological replicates in chemically defined Aquil artificial seawater media. *Fragilariopsis cylindrus* cultures were subjected to six different treatments including (1) optimal growth (+4 °C, nutrient replete, 24 h light at 35 μmol photons m^−2^ s^−1^), (2) freezing temperatures (−2 °C, nutrient replete, 24 h light at 35 μmol photons m^−2^ s^−1^), (3) elevated temperatures (+11 °C, nutrient replete, 24 h light at 35 μmol photons m^−2^ s^−1^), (4) elevated carbon dioxide (+4 °C, 1,000 ppm CO_2_, 24 h light at 35 μmol photons m^−2^ s^−1^), (5) low iron (+4 °C, -Fe, 24 h light at 35 μmol photons m^−2^ s^−1^), (6) prolonged darkness (+4 °C, nutrient replete, 7 d darkness).

*F. cylindrus* stock cultures from exponential growth phase were used to inoculate 2 l experimental batch cultures with an initial cell count of 50,000 cells ml^−1^. During experimental treatments (except elevated CO_2_ treatment), cultures were bubbled with filtered ambient air (Swinnex unit equipped with 25 mm Whatman GF/F filter) passed through milliQ-H2O and manually shaken before subsampling to ensure sufficient CO_2_ supply and mixing. Subsamples were taken on a daily basis throughout the experiments to determine physiological parameters including specific growth rate and maximum quantum yield of photosystem II (F_v_/F_m_) as a proxy for cell fitness^14^. Cell counts were determined using automated cell counting with a Multisizer 3 particle counter (Beckman Coulter, Brea, CA, USA) equipped with a 100 μm aperture capillary.

Whilst the experimental treatment of *F. cylindrus* with elevated carbon dioxide was instantly applied to cell cultures, experimental cultures grown under prolonged darkness, freezing temperatures and elevated temperatures were first grown to early exponential phase at optimal growth conditions before shifting to the final experimental conditions. These experimental treatments were initiated during early exponential phase when cultures had a cell density of approximately 300,000 cells per ml. For low iron treatments, *F. cylindrus* was grown in iron-free Aquil media that had been passed through a Chelex cation exchange column (Chelex 100 Resin, biotechnology grade sodium form, 100–200 dry mesh size, 150–300 μm wet bead size, Bio-Rad Laboratories, Hercules, CA, USA). Cells from iron-replete stock cultures were transferred into iron-free Aquil media and allowed to grow for several days prior to experimentation to ensure iron limitation as performed previously^15^. Preparation of iron-free Aquil media and handling of low iron cultures were carried out using standard trace metal clean techniques as described for trace metal studies^11,16,17^. Accordingly, 2 l aliquots of Aquil seawater were supplemented with macronutrients (NO_3_, PO_4_ and Si(OH)_4_ in accordance with Aquil medium concentrations), passed through a Chelex cation exchange column, filter-sterilised (nitrocellulose membrane filter, 47 mm 0.22 μm GSWP, Millipore, MA, USA) and placed into 10% hydrochloric acid-cleaned, milli-Q H_2_O-rinsed 2.5 l polycarbonate bottles. Trace metal concentrations were buffered using 100 μmol l^−1^ of ethylenediaminetetraacetic acid (EDTA), which reacts with metal ions (including Fe^3+^) to metal chelates that are not directly available to phytoplankton, rendering potential iron contaminations insignificant. Dispensed chelexed and filter-sterilised Aquil seawater was supplemented with filter-sterilised (25 mm 0.2 μm syringe filter) EDTA-trace metals (minus iron) and vitamins (B_12_, thiamine and biotin), and allowed to equilibrate chemically overnight at final growth conditions before inoculation of cells. Experimental cultures were sampled for RNA preparations when they reached mid-exponential phase (~500,000 cells ml^−1^) after 5–10 days of acclimation to the experimental treatment by gentle filtration of cultures (~300 psi vacuum pressure) onto 1.2 μm membrane filters (Isopore membrane, Millipore, MA, USA), placement in 2 ml cryogenic centrifuge tubes and flash-freezing in liquid nitrogen. Finally, the limiting effect of experimental treatments on *F. cylindrus* was confirmed according to La Roche *et al.*^18^, which is based on addition of the limiting nutrient to reconstitute optimal growth conditions leading to the recovery of physiological parameters that are depressed by the experimental treatment.

### RNA preparation

Total RNA was extracted using a guanidinium thiocyanate-phenol chloroform extraction according to Chomczynski & Sacchi^19^ using a TRI Reagent (Sigma-Aldrich, St Louis, MO, USA) protocol^20^. All samples were treated with DNase I (Quiagen, Hilden, Germany) for 1 h at 37 °C and RNA was purified using RNeasy MinElute Cleanup Kits (Quiagen, Hilden, Germany) according to the manufacturer’s instructions. RNA was checked for DNA contamination using no reverse transcriptase controls in qRT-PCR experiments. A detailed description of qRT-PCR experiments including primer sequences and reaction conditions can be found in (ref. 20) and (ref. 21).

### Library preparation and sequencing

RNA-seq library preparation and Illumina sequencing was performed by Edinburgh Genomics (Edinburgh, UK). Libraries were constructed according to the RNA-seq Sample Prep Kit (Illumina, San Diego, CA, USA) and cDNA synthesis was performed with random hexamers and reverse transcriptase. Subsequently, samples were sequenced in high-throughput manner to obtain short sequence reads using an Illumina HiSeq 2000 platform. Sequencing was conducted according to the Illumina TruSeq RNA Sequencing protocol. Triplicate samples of *F*. *cylindrus* grown under six different experimental conditions were run in a single lane of a flowcell using multiplex DNA barcodes, generating paired-end reads of 101 bases length (Data Citation 2).

### RNASeq read mapping

We mapped each of the RNA-seq experiment to the *FALCON* assembly using STAR 2.3.0 (ref. 22) alignment with standard settings using the command.

STAR --genomeDir./genome --runThreadN 4 --readFilesIn -outFileNamePrefix.

### Code availability

Code is available at github.com/Paajanen/FC_scientific_data including the parameter files for generating the assembly, and the annotation.gtf file.

## Data Records

The PacBio genome assembly and sequence data (Data Citation 1) has been deposited at DDBJ/EMBL/GenBank under accession number PRJEB15040. *Fragilariopsis cylindrus* RNA-seq data (Data Citation 2) are available in the ArrayExpress database (http://www.ebi.ac.uk/arrayexpress) under accession number E-MTAB-5024. The *F. cylindrus* fosmids (Data Citation 3) are available in GenBank under accession numbers AC275650-AC275663.

## Technical Validation

### DNA sample quality

Bacterial contaminations were eliminated by subjecting cultures to ampicillin (50 μg ml^−1^) and chloramphenicol (1 μg ml^−1^) for 3 days. Fluorescence microscopy combined with 4′,6-diamidino-2-phenylindole (DAPI) fluorescence nucleic acid staining was used to confirm axenic cultures before DNA extraction and sequencing.

### RNA quality

Purity of RNA was checked using a NanoDrop spectrophotometer (Thermo Fisher Scientific, Waltham, MA, USA) and RNA integrity was assessed using 2% denaturating formaldehyde gels and an Agilent 2100 Bioanalyzer electrophoresis system (Agilent, Santa Clara, CA, USA).

### Read data quality control

**Figure 3:**
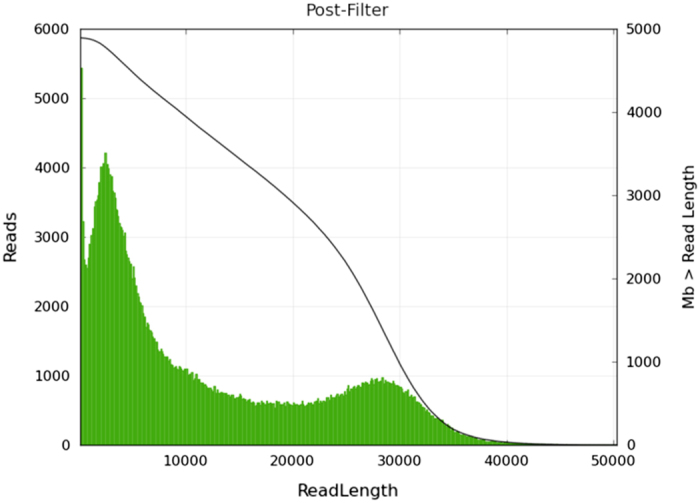
PacBio read length for 20 kb library. Distributions of read length from the PacBio sequencer.

We assessed that the read quality was long enough for assembly by observing the read length distribution of the 20 kb PacBio sequencing library (Fig. 3).

### Assembly validation

#### Assembly size

The haploid assembly resulted in primary contigs from which we deduced a genome size of 59.7 Mb. The genome size was independently estimated as 57.9 (±16.9) Mb using RT-qPCR^1^.

#### Local accuracy

We assessed the accuracy of this assembly using Sanger finished haplotyped fosmids (Data Citation 3), which we aligned with bwa 0.7.12 (ref. 23) using the command bwa mem -x pacbio.

All fosmids aligned to the assembly. One of the fosmids aligned perfectly over 43,010 bp with no SNPs or indels, while the worst fosmid aligned for 14 kb region. The lengths of the alignments were calculated using samtools 1.3 (ref. 9) and awk^24^ to count positions that were mapped.

The counts of indels and SNPs in each of the fosmids were calculated manually and are summarized in Table 3. We calculated the percentage accuracy by adding the number of SNPs and indels and divided it by the length of alignment.

#### Mapping the reads back to the assembly

We created circular consensus (ccs) reads from the 4 kb insert size library using the SMRTANALYSIS 2.3.0p5 pipeline and a cut-off of 500 bp as minimum read length with the command

smrtpipe.py --params=ReadsOfInsert.params.xml xml:input.xml > smrtpipe.log

where input.xml was created from a file input.fofn containing paths to the h5-files from the SMRTcells from the 4 kb libraries fofnToSmrtpipeInput.py input.fofn > input.xml and ReadsOfInsert.params.xml (github.com/paajanen/FC_scientific_data). The circular consensus reads had an average number of 10 passes, and the mean read quality of the inserts was >0.9862 for all four SMRT cells. The total number of reads was 119,511 with a mean of 2,429 bp. The total number of bases of ccs reads was 290,305,468 bp, resulting in a coverage of 4.8x over a genome of size ca. 60 Mb. We aligned these reads to the primary and alternate contigs of the assembly using bwa 0.7.12 (ref. 23) with the command bwa mem -x pacbio. The coverage was calculated using samtools 1.3 (ref. 9) and awk^24^ with

samtools depth -aa falcon_aligned_roi.bam > falcon_aligned_roi.coverage

awk '{for(i=1;i<=NF;i++) {sum[i] += $i; sumsq[i] += ($i)^2}} END {for (i=1;i<=NF;i++) {print "%f %f \n", sum[i]/NR, sqrt((sumsq[i]-sum[i]^2/NR)/NR)}}'

done

After using grep to find the right scaffolds, the coverage was calculated.

The mean (±s.e.m.) coverage of the primary and alternate contigs on scaffold 000002F was 4.59 (±2.71) and 2.51 (±1.99).

### Quality of annotation

We searched the gtf annotation file with the custom python script coverage_identity.py (github.com/paajanen/FC_scientific_data)

and verified that 24,635 out of the 26,418 transcripts (93.3%) from the JGI annotation map to the FALCON assembly with at least 95% coverage and 95% identity.

### Allelic pairs in the FALCON assembly

Using the 24,535 transcripts from the JGI annotation that map to the FALCON assembly, we updated the list of allelic pairs identified in the ARACHNE assembly^1^, using the custom script SD.find_allelic_pairs.py (github.com/paajanen/FC_scientific_data). Out of the 6,071 allelic pairs identified in the ARACHNE assembly 5,400 (89%) are found in the FALCON assembly. A total of 1,894 of the 5,400 allelic pairs in the FALCON assembly map in to alternate haplotypes, while the rest are ambiguous, and are either paralogs or located on smaller scaffolds that are not easily distinguished as alternate haplotypes. Additionally, we identified 1,615 transcripts on alternate haplotype contigs that are not assigned as allelic pairs in the ARACHNE assembly. Some of these have identical copies at the corresponding location of the primary contig, possibly because polishing the assembly has polished out the differences. Lists of JGI protein identifiers for (1) confirmed alternate allelic pairs in the ARACHNE assembly, (2) transcripts that have alternate alleles in both, ARACHNE and FALCON assembly, and (3) new possible transcripts with alternate allele are in Supplementary Table 1.

### RNA-seq data validation

#### Metrics of the RNA-seq dataset

A total of 421 million reads were generated, with an average of 3.8 million read pairs per sample (elevated temperature, elevated carbon dioxide, darkness, optimal growth, low iron, low tempertature). The reads were analysed with FasQC software, which did not flag any sample as bad quality. The average read quality was 36 (mean) and 37 (median) for each sample. The samples had negligible amounts of adapter residues. The read length was 101 bp.

#### Alignment validation

The percentage of reads aligning uniquely varies from 68 to 74%, depending on the experiment (Table 1). We also counted the number of mapped reads for each experiment with htseq version 0.6.1 (ref. 25) using the command htseq-count --mode=intersection-nonempty --stranded=no --type=exon Aligned.sam annotation.gtf.

## Usage Notes

The genome assembly (ref. 1) sequencing reads described in this work were intended to confirm the published assembly^1^ and to clarify the diverged haplotypes. We have not annotated the PacBio FALCON assembly from scratch, but rather used the annotation of the ARACHNE assembly produced by JGI to map to the FALCON assembly. Therefore, the ARACHNE assembly based on Sanger sequencing might be more suitable for detailed genetic studies, while the FALCON assembly should be used for longer range information and precise location of allelic diversity. RNA-seq data can be used to map to either assembly based on individual needs.

## Additional Information

**How to cite this article:** Paajanen, P. *et al.* Building a locally diploid genome and transcriptome of the diatom *Fragilariopsis cylindrus*. *Sci. Data* 4:170149 doi: 10.1038/sdata.2017.149 (2017).

**Publisher’s note:** Springer Nature remains neutral with regard to jurisdictional claims in published maps and institutional affiliations.

## Supplementary Material



Supplementary Table 1

Supplementary Table 2

Supplementary Table 3

## Figures and Tables

**Table 1:** General description and mapping statistics for *Fragilariopsis cylindrus* RNA-seq data set.

**Experimental factor**	**Sample code**	**Replicate**	**Sample ID [ENA run accession]**	**Insert size**	**Total read count**	**Unique mapping (% of total)**	**Ambiguous mapping (% of total)**	**Unmapped reads (% of total)**
elevated temperature	HEAT1	r1	ERR1580555	229	4429184	3148542 (71%)	1044963 (24%)	235679 (5%)
elevated temperature	HEAT2	r2	ERR1580556	211	4126604	2846691 (69%)	1016443 (25%)	263470 (6%)
elevated temperature	HEAT3	r3	ERR1580557	233	4497930	3008589 (67%)	1161777 (26%)	327564 (7%)
elevated carbon dioxide	CO2_1	r1	ERR1580558	236	4604350	3167275 (69%)	1210586 (26%)	226489 (5%)
elevated carbon dioxide	CO2_2	r2	ERR1580559	200	3266562	2209951 (68%)	869628 (27%)	186983 (6%)
elevated carbon dioxide	CO2_3	r3	ERR1580560	240	4820992	3333628 (69%)	1252195 (26%)	235169 (5%)
darkness	DARK1	r1	ERR1580561	199	3471467	2516325 (72%)	725945 (21%)	229197 (7%)
darkness	DARK2	r2	ERR1580562	203	4472910	3251588 (73%)	975633 (22%)	245689 (5%)
darkness	DARK3	r3	ERR1580563	218	4774704	3468462 (73%)	1017985 (21%)	288257 (6%)
optimal growth	CTRL1	r1	ERR1580564	218	2481346	1692631 (68%)	665392 (27%)	123323 (5%)
optimal growth	CTRL2	r2	ERR1580565	235	3413265	2348061 (69%)	906511 (27%)	158693 (5%)
optimal growth	CTRL3	r3	ERR1580566	254	2969725	2087259 (70%)	751742 (25%)	130724 (4%)
low iron	FE1	r1	ERR1580567	236	3224916	2351646 (73%)	737170 (23%)	136100 (4%)
low iron	FE2	r2	ERR1580568	231	3070321	2237270 (73%)	700692 (23%)	132359 (4%)
low iron	FE3	r3	ERR1580569	239	2961818	2178101 (74%)	645655 (22%)	138062 (5%)
freezing	COLD1	r1	ERR1580570	261	4639725	3198832 (69%)	1159928 (25%)	280965 (6%)
freezing	COLD2	r2	ERR1580571	233	3674305	2564764 (70%)	925973 (25%)	183568 (5%)
freezing	COLD3	r3	ERR1580572	232	3932382	2745134 (70%)	980834 (25%)	206414 (5%)

**Table 2:** Statistics of the *FALCON* assembly.

**Names**	**No contigs**	**N50**	**Mean**	**Max**	**Length**
Primary	745	245 kb	352 kb	1.26 Mb	59.7 Mb
Alternate	288	41 kb	47 kb	127 kb	9.1 Mb

**Table 3:** Accuracy of Fosmid alignments to the polished Falcon assembly.

**Fosmid name**	**Length of fosmid (bp)**	**Length of alignment to assembly (bp)**	**Number of Snps**	**Number of Indels**	**% accuracy**
4082860	31,629	31,509	162	97	99.2
4082861	34,510	34,352	77	114	99.4
4082862	34,747	34,714	20	29	99.9
4082863	35,759	33,456	3	14	99.9
4082864	35,086	35,084	0	2	99.9
4082865	43,010	43,010	0	0	100
4082866	31,655	31,289	324	66	98.8
4082867	34,904	29,133	99	135	99.2
4082868	36124	14,738	38	7	99.7
4082870	35,909	35,839	53	40	99.8
4082871	32,224	32,223	0	1	99.9
4082872	36,274	36,232	22	30	99.9
4082873	30,696	29,409	387	87	98.5
The percentage of accuracy was calculated by taking the sum of snps and indels and dividing it by the length of alignment.					
